# Factors associated with Successful Responses to Ganglion Impar Block: A Retrospective Study

**DOI:** 10.7150/ijms.60962

**Published:** 2021-06-11

**Authors:** Chan-Sik Kim, Kyounghwan Jang, Jeong-Gil Leem, Jin-Woo Shin, Doo-Hwan Kim, Seong-Soo Choi

**Affiliations:** Department of Anesthesiology and Pain Medicine, Asan Medical Center, University of Ulsan College of Medicine, Seoul, Republic of Korea

**Keywords:** Ganglion impar block, Coccydynia, Perineal pain, Cancer pain

## Abstract

**Background:** The ganglion impar (ganglion of Walther) block has been used to manage coccygeal and perineal (perianal and genital) pain due to both benign and malignant causes. However, the factors associated with successful responses to ganglion impar block are unknown. Therefore, in the present study, we aimed to identify the independent factors associated with successful responses to ganglion impar block in patients with chronic pain in coccygeal and perineal regions.

**Methods:** From January 2008 to December 2017, we performed a retrospective review of 106 patients who underwent ganglion impar block. Patients were considered successful responders if they reported a decrease of more than 50% or 4 points on the 11-point (0 = no pain and 10 = worst possible pain) numerical rating scale 1 month after the procedure, while others were considered non-responders. Logistic regression analysis was performed to identify factors independently associated with successful responses at 1 month after the procedure.

**Results:** Multivariable logistic regression analysis showed that cancer-related causes were significantly associated with successful responses at 1 month after ganglion impar block (odds ratio = 2.60, 95% confidence interval = 1.05 to 6.43, *P* = 0.038).

**Conclusion:** Ganglion impar block may be more effective in cancer-related pain than pain due to benign causes.

## Introduction

The ganglion impar (ganglion of Walther) block was originally described in the treatment of sympathetically mediated cancer pain involving the perineum 1. Since then, it has also been used to alleviate coccygeal and perineal (perianal and genital) pain due to both benign and malignant causes 2, 3.

Previous studies have shown the effectiveness of ganglion impar block 4-6 and radiofrequency thermocoagulation 7 in patients with chronic coccydynia. In addition, ganglion impar block 8, 9 and radiofrequency thermocoagulation 10 for perianal pain may also be effective in decreasing pain intensity. Furthermore, there are several case reports which revealed that ganglion impar block may be useful in managing genital pain 11-13.

Although the effectiveness of ganglion impar block has been shown in prior studies 4, 5, 14, the factors associated with successful responses have not been fully evaluated. To prevent unnecessary expenses and harm to the patient, proper patient selection for ganglion impar block is needed. Therefore, in the present study, we aimed to identify factors independently associated with successful responses to ganglion impar block in patients with chronic pain in coccygeal and perineal regions.

## Materials and methods

### Patients

This retrospective study was performed at the pain clinic in our institution. This study was performed in accordance with the Declaration of Helsinki and the study protocol was approved by the institutional review board of Asan Medical Center, Seoul, Korea (approval number: 2018-0279). Informed consent was waived owing to the retrospective nature of the study. We searched our institution's Information Technology of Service Management (ITSM) system between January 2008 and December 2017 with the terms “ganglion impar block.” The inclusion criteria were as follows: 1) age 19-80 years, 2) chronic pain in coccygeal and perineal regions over 3 months, 3) received ganglion impar block. We included the patients who received ganglion impar block, not ganglion impar neurolysis. We excluded the patients who had any of the following conditions: 1) incomplete medical record, 2) loss to follow-up, 3) technical failure of block.

### Procedure: Ganglion Impar Block

**Figure 1:**
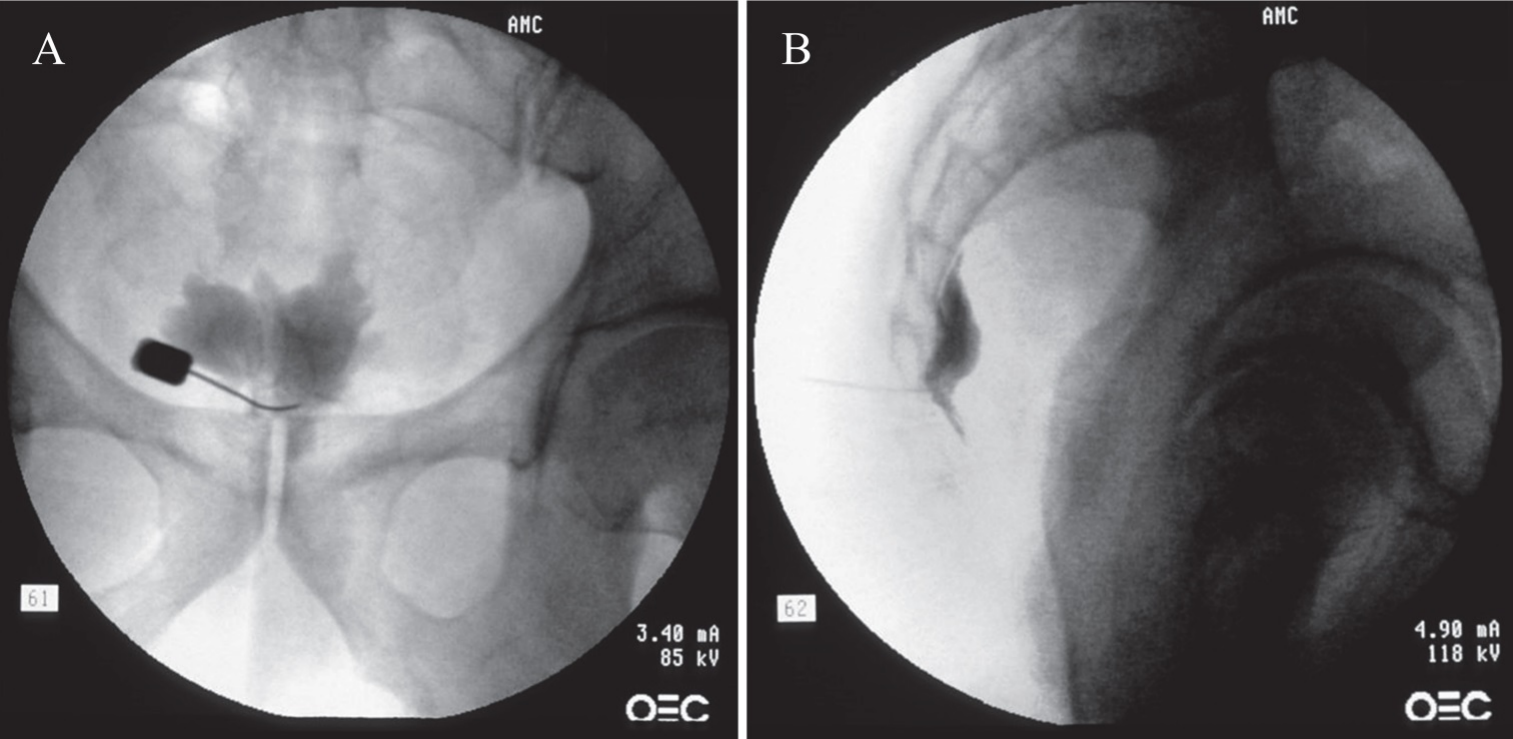
Fluoroscopic images of the ganglion impar block: transsacrococcygeal approach. (A) Anteroposterior view. The contrast flow in the midline at the upper coccyx. (B) Lateral view. The contrast flow just anterior to the upper coccyx showing comma sign.

All procedures were performed on an outpatient basis. After informed and written consent, the procedure was performed using a transsacrococcygeal approach 15. Medications or sedatives were not used before the procedure to prevent incidental neural damage and allow the patient's cooperation during the procedure. The patient was placed on the table in the prone position with a pillow under the lower abdomen. The intergluteal crease, anus, and surrounding area were prepared and draped in a sterile fashion. The procedure was performed under fluoroscopic guidance. After a true lateral image was obtained, skin and soft tissue were anesthetized with 1% lidocaine over the sacrococcygeal disc after identifying the disc in the lateral projection. A 25-G Quincke spinal needle (TaeChang Industrial Co., Ltd., Gonju-si, Chungcheongnam-do, Korea) was inserted under fluoroscopic guidance through the sacrococcygeal disc. The needle placement was confirmed by the injection of 0.2-0.5 mL of a contrast medium (Omnipaque; Nycomed Imaging, Oslo, Norway) (Fig. 1). After negative aspirate, 1-3 mL of local anesthetic (0.125-0.25% bupivacaine) was injected.

### Outcome Evaluation and Factors Associated with Successful Responses

The outcome evaluation was performed at baseline and 1 month after the procedure. For the outcome assessment, pain intensity measured using an 11-point (0 = no pain and 10 = worst possible pain) numerical rating scale (NRS-11) was reviewed from each patient's medical record 16. Additionally, baseline characteristics such as age, sex, body mass index, underlying diseases, location of pain, and cause of pain were obtained for analysis. Patients were considered successful responders if they reported a decrease of more than 50% or 4 points on the NRS-11 1 month after the procedure, while others were considered non-responders 17.

### Statistical Analysis

Data were analyzed using R version 4.0.2 (R Foundation for Statistical Computing, Vienna, Austria). Continuous demographic data from the non-responders and successful responders were compared using Student's t-test or the Wilcoxon rank-sum test and documented as means with standard deviations or medians with interquartile ranges, as appropriate. Categorical demographic data were compared using the chi-squared test or Fisher's exact test. By using logistic regression, the factors associated with successful responses at 1 month after ganglion impar block were analyzed. The inclusion of variables in the logistic regression analysis to identify the independent factors associated with successful responses was based on biological plausibility, clinical importance, and statistical considerations. The goodness-of-fit of the model was assessed with the Hosmer-Lemeshow test. A value of *P* < 0.05 was considered statistically significant.

## Results

**Figure 2:**
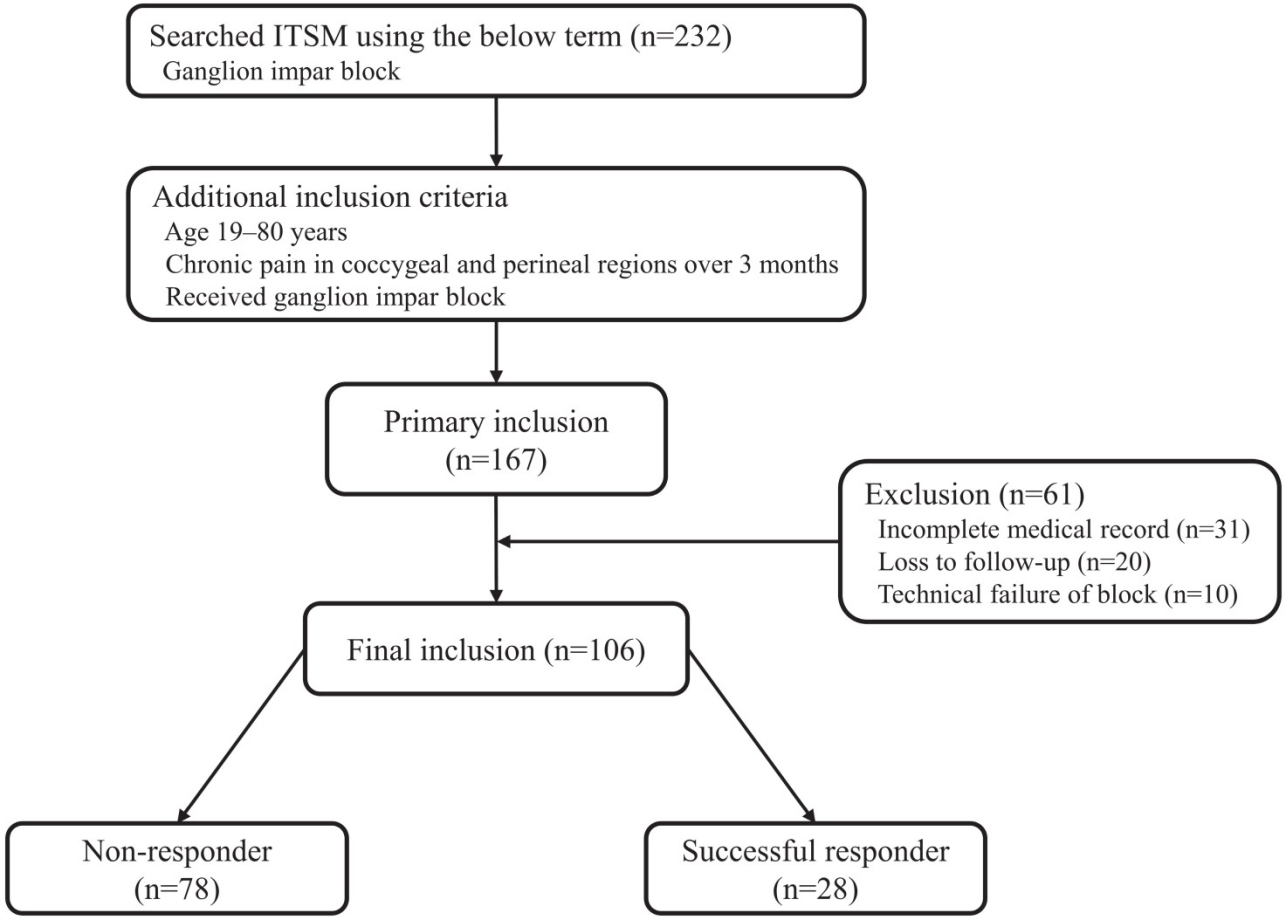
Flowchart of the present study. ITMS: information technology of service management.

As a result of the ITSM search, we found 232 patients who received ganglion impar block. A total of 167 patients met the inclusion criteria. Sixty-one patients were excluded due to incomplete medical record, loss to follow-up, or technical failure of block. Therefore, a total of 106 patients were included in the analysis. One month after the procedure, 28 patients reported a decrease of more than 50% or 4 points on the NRS-11 and were considered successful responders in this study. The 78 remaining patients who reported a decrease of less than 50% or 4 points on the NRS-11 were considered non-responders (Fig. 2).

The demographic characteristics of non-responders and successful responders at 1 month after ganglion impar block are summarized in Table 1. Causes of pain were significantly different between the groups (*P* = 0.044). Pelvic or perineal organ cancer, including urogenital organ cancer, colorectal cancer, and metastasis of non-pelvic and non-perineal cancer were major causes in the successful responder group. Conversely, major causes of pain in the non-responder group were those other than cancer, including trauma or anatomical abnormality, non-cancer pelvic operation, and unknown causes. There were no significant differences between the groups in other baseline characteristics.

As shown in Table 2, pre-procedural NRS-11 scores were not significantly different between the groups (non-responder, 7.0 (6.0-8.0); successful responder, 6.0 (4.5-8.0); *P* = 0.164). Post-procedural NRS-11 scores of pain intensities in non-responders and successful responders were 7.0 (5.0-8.0) and 2.0 (1.0-3.0), respectively (*P* < 0.001).

**Figure 3:**
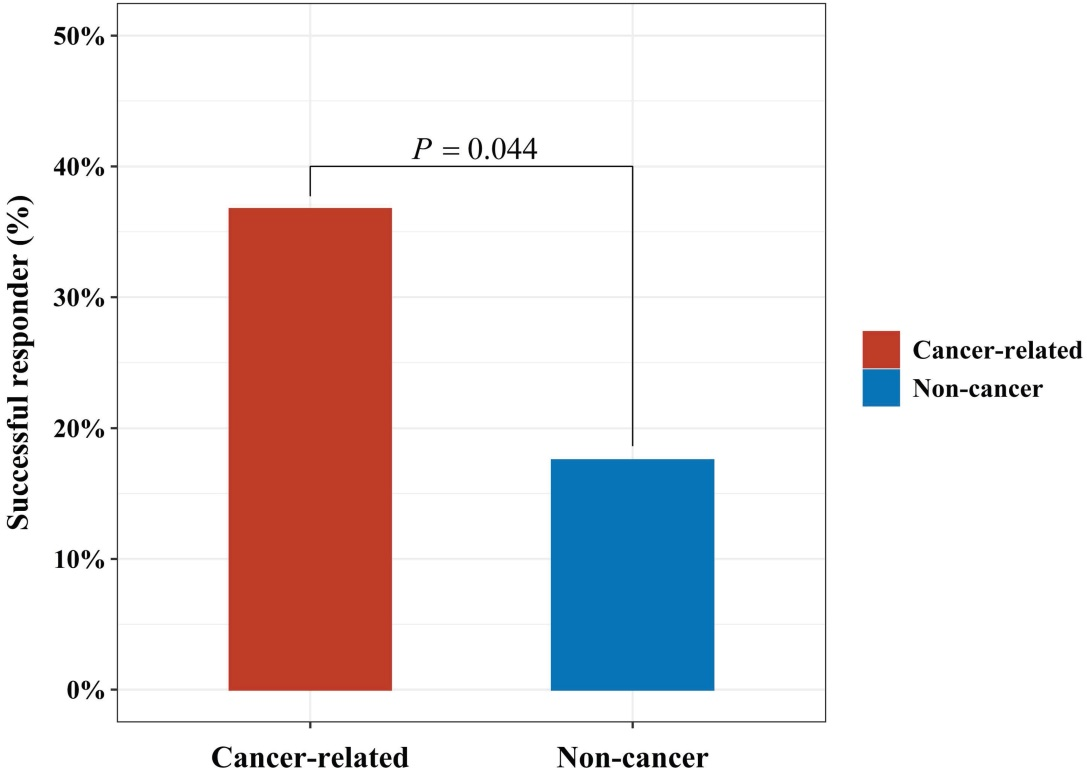
The proportion of successful responder in cancer-related and non-cancer-related causes.

In addition, the proportion of successful responder in cancer-related and non-cancer-related causes were 36.7% and 17.5%, respectively (*P* = 0.044, Fig. 3).

**Figure 4:**
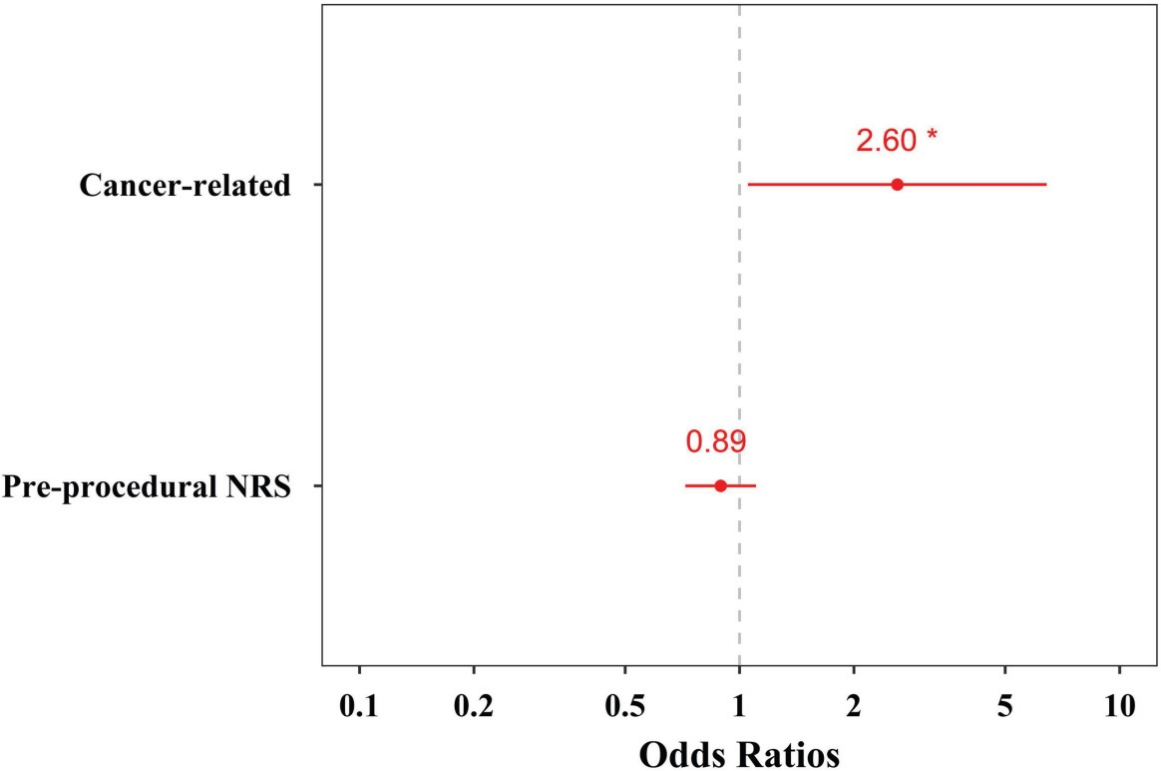
Odds ratio plot of the explanatory variables included in the multivariable logistic regression analysis for successful responses to ganglion impar block. The red dots and numbers represent actual odds ratios, and the red bars denote 95% confidence intervals.

Multivariable logistic regression analysis revealed that the cause related to pelvic or perineal organ cancer was significantly associated with successful responses at 1 month after ganglion impar block (odds ratio = 2.60, 95% confidence interval = 1.05 to 6.43, *P* = 0.038, Table 3, Fig. 4).

## Discussion

Despite the widespread use of ganglion impar block for managing coccygeal and perineal pain, the factors associated with successful responses have not been fully evaluated. The current study showed that cancer-related coccygeal and perineal pain was more responsive to ganglion impar block than pain due to benign causes.

The ganglion impar block was originally described by Plancarte et al. in 1990 to treat sympathetically mediated cancer pain involving the perineum 1. Since then, there have been only a few experimental or analytical studies evaluating the effectiveness of ganglion impar block. Notwithstanding the low-level evidence about the effectiveness of ganglion impar block, the literature describes the use of this block to alleviate coccygeal and perineal pain due to both malignant and benign causes 3, 14.

In this study, multivariable logistic regression analysis revealed that cancer-related causes were an independent factor associated with successful responses to ganglion impar block in patients with chronic pain in coccygeal and perineal regions. The ganglion impar is said to supply nociceptive and sympathetic fibers to the perineum and pelvic viscera, including the distal rectum, perianal region, distal urethra, vulva or scrotum, and the distal third of the vagina 18. Therefore, ganglion impar block may have been effective in pelvic and perineal organ cancer-related pain. On the other hand, in the case of non-cancer-related pain, there was a possibility that the pain may have been mediated by nerves other than ganglion impar, for example, pudendal nerve or genitofemoral nerve. The pudendal nerve carries sensory, motor, and sympathetic fibers to the distal aspect of the anal canal, perianal skin, vulva, vagina, clitoris, glans penis, and scrotum 19, 20. The spermatic cord, scrotum, labia majora, and mons pubis are innervated by the genital branch of genitofemoral nerve 21. Pudendal and genitofemoral neuralgia can cause pain in these cutaneous regions and may not respond to ganglion impar block. Therefore, in the present study, this may explain at least in part why the ganglion impar block was less effective in non-cancer-related pain than in pelvic or perineal visceral organ cancer-related pain.

Adas et al. 10 performed ganglion impar radiofrequency treatment for patients with chronic intractable perianal pain of various etiologies. Contrary to the present study, Adas et al. showed that malignant causes may be a risk factor for unsuccessful response to the ganglion impar radiofrequency ablation. They included only patients who had a prior positive response (at least 50% reduction by the visual analogue scale) in diagnostic ganglion impar block with local anesthetics. However, in the present study, all patients who received only ganglion impar block were included. Additionally, the definition of successful response (visual analogue scale of <4) and time for outcome evaluation (6 months after the procedure) were different from the present study. Moreover, Gunduz et al. 4 retrospectively analyzed 22 patients with chronic coccydynia in whom ganglion impar block was performed. They reported a success rate of 82%, while our results showed a success rate of 26%. A difference in study design may explain this inconsistency in success rates. Their study included patients with intractable coccydynia despite conservative treatment for at least 6 months and with no abnormalities on laboratory findings or imaging. In contrast, we included all patients who received ganglion impar block, regardless of pain location, response to previous conservative treatment, and abnormalities on laboratory findings or imaging. Therefore, these discrepancies are believed to be due to differences in the patient population included, definition of response, and time for outcome measurement after the procedure.

In our study, the proportion of successful responders in cancer-related causes was more than twice compared to that in non-cancer-related causes. However, 63.3% of patients with cancer-related pain still showed unsuccessful response to ganglion impar block. It is well-known that cancer-related pain is mediated by both somatic and visceral sensory fibers. Obviously, the present results suggested that ganglion impar block may not sufficiently interrupt somatic malignant painful stimulation, although it showed more effective response in patients with cancer-related pain than in those with non-cancer-related pain. In addition, because the superior hypogastric plexus also carries visceral nociceptive stimuli from pelvic organs 22, pelvic organ cancer-related pain in non-responsive patients may have been mediated by the superior hypogastric plexus as well as the ganglion impar. This may also explain why some cancer-related pain did not respond to ganglion impar block. Ahmed et al. 23 reported their results of a series of patients with pelvic or perineal cancer-related pain who underwent combined neurolytic superior hypogastric plexus block and ganglion impar block. They found that successful response, defined as lowering of the pre-procedural visual analogue scale by more than 50%, occurred in 66.6% of patients with pelvic or perineal cancer-related pain. Their finding suggested that ganglion impar block combined with superior hypogastric plexus block may provide more pain relief in pain due to pelvic or perineal organ cancer than ganglion impar block alone.

There were several limitations to this study. First, the definition of successful response in the present study somewhat differed from the ones used in previous studies. This may lead to different results. We defined successful response in the present study based on IMMPACT recommendations 17. According to the IMMPACT recommendations, a decrease of ≥4 points or ≥50% on the NRS-11 in pain intensity appears to represent a substantial (very much improved) change in pain, which patients have also considered treatment success or satisfactory improvement. Second, the present study was a retrospective study with a small sample size, which could weaken the result of this study. However, we performed various statistical analyses, including multivariable logistic regression analysis. Hence, we believe that these methods may improve the statistical significance of our results. Third, we evaluated patients' responsiveness only by pain measured with the NRS-11 because there was a lack of data about other core domains for chronic pain clinical trials. A further study evaluating not only pain but also other core domains, including physical functioning, emotional functioning, and global rating of improvement, is required.

In conclusion, ganglion impar block may be more effective in cancer-related pain than pain due to benign causes. Therefore, ganglion impar block could be a treatment option for pain due to pelvic or perineal organ cancer.

## Figures and Tables

**Table 1:** Patient characteristics.

	Non-responder (n = 78)	Successful responder (n = 28)	*P* value
Age (years)	56.5 ± 13.2	58.8 ± 8.8	0.300
Sex (male/female)	29 (37.2%) / 49 (62.8%)	9 (32.1%) / 19 (67.9%)	0.805
BMI (kg/m^2^)	23.2 ± 4.1	23.5 ± 2.4	0.665
Diabetes	8 (10.3%)	2 (7.1%)	> 0.999
Hypertension	18 (23.1%)	6 (21.4%)	> 0.999
Pain location			0.721
Coccygeal	22 (28.2%)	8 (28.6%)	
Perianal	45 (57.7%)	14 (50.0%)	
Genital	8 (10.3%)	5 (17.9%)	
Other	3 (3.8%)	1 (3.6%)	
Cause			0.044
Pelvic or perineal organ cancer (including metastasis)	31 (39.7%)	18 (64.3%)	
Other than cancer (including unknown causes)	47 (60.3%)	10 (35.7%)	

Data are expressed as mean ± standard deviation or numbers (%). BMI: body mass index.

**Table 2:** Procedural characteristics.

	Non-responder (n = 78)	Successful responder (n = 28)	*P* value
Pre-procedural NRS-11	7.0 (6.0-8.0)	6.0 (4.5-8.0)	0.164
Post-procedural NRS-11	7.0 (5.0-8.0)	2.0 (1.0-3.0)	< 0.001

Data are expressed as median (interquartile range). NRS-11: 11-point numerical rating scale.

**Table 3:** Logistic regression analysis of the factors associated with successful responses after ganglion impar block.

	Univariable			Multivariable		
Parameters	OR	95% CI	*P* value	OR	95% CI	*P* value
Age (years)	1.02	0.98-1.05	0.385			
Sex						
Male (reference)	1.00					
Female	1.25	0.50-3.12	0.634			
Cause						
Non-cancer (reference)	1.00			1.00		
Cancer-related	2.73	1.11-6.69	0.028	2.60	1.05-6.43	0.038
Pre-procedural NRS-11	0.87	0.70-1.08	0.197	0.89	0.72-1.10	0.296

OR: odds ratio; CI: confidence interval; NRS-11: 11-point numerical rating scale.

## Abbreviations

BMI: body mass index
CI: confidence interval
ITMS: information technology of service management
NRS-11: 11-point numerical rating scale
OR: odds ratio
